# Intralesional Epidermal Growth Factor and Its Association with Reamputation and Level-Up Amputation in Diabetic Foot Patients with Post-Amputation Flap Necrosis: Clinical Outcomes and Macrophage Dynamics

**DOI:** 10.3390/medicina62091800

**Published:** 2026-09-18

**Authors:** Ali Murat Basak, Kubra Canarslan Demir, Adile Begum Bahcecioglu, Munire Kubra Ozgok Kangal, Yasin Hatipoglu, Hamdullah Yanik, Omer Levent Karadamar, Yasin Gulap, Mehmet Mert Hidiroglu, Simay Akyuz, Kerim Bora Yilmaz

**Affiliations:** 1 Department of Orthopedics and Traumatology, Gülhane Training and Research Hospital, University of Health Sciences, Ankara 06010, Türkiye; muratalibasak@gmail.com (A.M.B.); leventkaradamar@gmail.com (O.L.K.); 2 Department of Undersea and Hyperbaric Medicine, Gülhane Training and Research Hospital, University of Health Sciences, Ankara 06010, Türkiye; drcanarslan@hotmail.com (K.C.D.); kubra_ozgk@hotmail.com (M.K.O.K.); 3 Department of Endocrinology and Metabolism, Gülhane Training and Research Hospital, University of Health Sciences, Ankara 06010, Türkiye; begumbahceci@hotmail.com; 4 Department of General Surgery, Gülhane Training and Research Hospital, University of Health Sciences, Ankara 06010, Türkiyeygulap@gmail.com (Y.G.); merthidiroglu28@gmail.com (M.M.H.); 5 Department of Medical Biology, Gülhane Faculty of Medicine, University of Health Sciences, Ankara 06010, Türkiye; yanikhamdullah@gmail.com; 6 Gülhane Faculty of Nursing, University of Health Sciences, Ankara 06010, Türkiye; 7 Department of Molecular Surgical Oncology, Gülhane Institute of Health Sciences, University of Health Sciences, Ankara 06010, Türkiye

**Keywords:** diabetic foot, epidermal growth factor, limb salvage, reamputation, amputation, flap necrosis, wound healing, wound immunophenotyping, diabetic foot infection

## Abstract

*Background and Objectives*: Post-amputation flap necrosis in diabetic foot patients is associated with a high risk of reamputation, level-up amputation, and limb loss. Intralesional epidermal growth factor (iEGF) has been shown to promote wound healing in diabetic foot ulcers; however, its role in the management of post-amputation tissue defects and flap necrosis remains unclear. This study aimed to evaluate the association between iEGF use and clinical outcomes in diabetic foot patients with post-amputation flap necrosis and to characterize longitudinal changes in wound macrophage-associated cell populations. *Materials and Methods*: This single-center observational cohort study, with retrospective identification of eligible patients and longitudinal follow-up, included 164 diabetic foot patients who developed flap necrosis, wound-healing disorders, or tissue defects following transmetatarsal, Lisfranc, Chopart, or below-knee amputations. Eighty-four patients received an iEGF-containing multidisciplinary limb-salvage strategy, while 80 patients received a multidisciplinary limb-salvage strategy without iEGF. The primary endpoint was treatment success, defined as preservation of the original amputation level without reamputation or proximal level-up amputation, together with satisfactory wound healing at the last available clinical follow-up. Secondary endpoints included mortality during follow-up and, in a flow cytometry sub-study of 12 iEGF-treated patients with available paired wound samples, changes in CD38^+^ and CD209^+^ cell proportions within the CD45^+^CD14^+^ macrophage-enriched population in paired wound-tissue samples. *Results*: Overall treatment success was 68.3% (112/164), and the observed crude cumulative mortality proportion was 21.3% (35/164). Treatment success was higher in the iEGF group than in the control group (95.2% [80/84] vs. 40.0% [32/80], *p* < 0.001), whereas the observed crude cumulative mortality proportion was lower (2.4% [2/84] vs. 41.3% [33/80], *p* < 0.001). In the flow-cytometry sub-study (*n* = 12), CD38^+^ cell proportions within the CD45^+^CD14^+^ macrophage-enriched population increased from 10.8% to 20.3%, whereas CD209^+^ cell proportions increased from 14.5% to 25.7% in paired samples collected before and after the iEGF treatment cycle (both *p* < 0.001). *Conclusions*: In this observational cohort of high-risk diabetic foot patients with post-amputation flap necrosis, wound-healing disorders, or tissue defects, iEGF administered as part of a multidisciplinary limb-salvage strategy was associated with higher treatment success, while a lower crude cumulative mortality proportion was also observed in the iEGF group. The mortality difference represents a crude cumulative comparison and should not be interpreted as evidence of a time-adjusted or treatment-associated survival benefit. Given the non-randomized treatment allocation, differences in vascular management and adjunctive therapies, and the potential for residual confounding, these findings should be interpreted as associative rather than causal. The paired tissue substudy demonstrated pre–post changes in CD38+ and CD209+ cell populations; these exploratory findings do not establish macrophage polarization or a causal biological mechanism.

## 1. Introduction

Diabetic foot disease is a major cause of lower-extremity amputation and is associated with substantial morbidity, mortality, loss of mobility, and impaired quality of life [1]. With the global increase in diabetes prevalence and life expectancy, the burden of diabetic foot ulcers (DFUs) continues to rise and remains a major public health challenge [2]. It is estimated that a diabetes-related lower-extremity amputation occurs every 30 s worldwide, with nearly 85% of cases preceded by a foot ulcer that progresses to infection, gangrene, and ultimately limb loss [3,4].

Infection is present in approximately half of DFU cases at the time of diagnosis and plays a pivotal role in disease progression. Once established, infection may rapidly extend to deep soft tissues and bone, leading to extensive tissue destruction [5]. In advanced stages, particularly in PEDIS stage 3–4 infections, the clinical course may become aggressive and life-threatening, sometimes resembling necrotizing soft tissue infections characterized by rapid progression, vascular thrombosis, and systemic inflammatory response [5,6,7]. In this setting, delayed intervention significantly increases morbidity and mortality.

In patients with progressive infection, osteomyelitis, and fascial involvement, repeated surgical debridement and minor or major amputations are frequently required. However, beyond infection control, the principal aim of surgical treatment is preservation of a functional limb. Major amputations, particularly above-ankle procedures, are associated with increased mortality, reduced mobility, metabolic deterioration, and diminished quality of life [8,9]. Therefore, limb salvage and maintenance of maximum foot surface area are of critical importance, especially in elderly patients in whom even limited preservation of plantar support may significantly facilitate mobilization.

In ischemic or infected diabetic feet, partial foot amputations such as Lisfranc or Chopart procedures are commonly performed using local flaps. However, these procedures are associated with high rates of postoperative complications, particularly flap necrosis, wound dehiscence, and surgical site infection. Compared with traumatic or oncologic amputations, diabetic patients demonstrate higher rates of complications and reamputation. Reported reamputation rates following forefoot and midfoot amputations range between 28% and 43%, and the development of flap necrosis markedly increases the likelihood of proximal, or “level-up,” amputation [10,11,12,13].

Intralesional epidermal growth factor (iEGF; Heberprot-P, Havana, Cuba) has been used in diabetic foot management to enhance granulation tissue formation and promote wound closure [14]. By stimulating fibroblast proliferation, epithelialization, angiogenesis, and extracellular matrix remodeling, iEGF contributes to volumetric reduction in tissue defects, including those with exposed tendon or bone [15,16,17]. In addition to its local regenerative effects, recombinant EGF may influence the inflammatory milieu of the wound microenvironment, potentially facilitating the transition from a prolonged inflammatory phase to a reparative phase [18].

Although recombinant EGF has been investigated in conventional diabetic foot ulcers, evidence specifically addressing post-amputation flap necrosis and tissue defects remains limited. This represents a clinically distinct and high-risk setting in which failure of wound healing may directly lead to reamputation or progression to a more proximal amputation level. Accordingly, the novelty of the present study lies in evaluating iEGF within this specific post-amputation population rather than in reassessing its general wound-healing effects in conventional diabetic foot ulcers. Furthermore, data regarding treatment-associated changes in the cellular composition of the wound microenvironment in this setting remain limited. Therefore, the primary objective of this study was to evaluate the association between intralesional EGF treatment and treatment success in diabetic foot patients who developed flap necrosis, wound-healing disorders, or tissue defects following lower-extremity amputation. The primary endpoint was treatment success, defined as preservation of the original amputation level without the need for reamputation or proximal level-up amputation, together with satisfactory wound healing at the last available clinical follow-up. Secondary objectives were to evaluate mortality during follow-up and to investigate changes in CD38^+^ and CD209^+^ cell proportions within the CD45^+^CD14^+^ macrophage-enriched population in wound tissue following iEGF treatment.

## 2. Materials and Methods

This study was designed as a single-center observational cohort study with retrospective identification of eligible patients from institutional medical records and longitudinal follow-up. The retrospective cohort included patients treated during the clinical record period from January 2018 through December 2024. January 2018 represents the earliest date of the clinical records included in the cohort and does not represent the initiation date of the research study. Clinical outcome information available in the institutional records was followed through December 2025. The study was approved by the Clinical Research Ethics Committee of Gülhane Training and Research Hospital, University of Health Sciences on 24 November 2022 (approval no. 2022/157). Informed consent was obtained from patients receiving iEGF before treatment. Patients included in the flow-cytometry substudy also provided informed consent for wound-tissue sampling and subsequent biological analysis. iEGF was supplied through the hospital pharmacy as part of routine clinical care. Patients who underwent transmetatarsal, Lisfranc, or Chopart amputations in the Departments of Orthopedics and Traumatology and General Surgery during the study period and subsequently developed flap necrosis, wound-healing disorders, or tissue defects were identified from institutional clinical records and evaluated for eligibility. In addition, patients who were evaluated at our center for wound-healing impairment following below-knee surgery for diabetic foot were assessed according to the same eligibility criteria. At the time of evaluation, all included patients had clinical indications for extensive surgical debridement, reamputation, and/or level-up amputation, reflecting a high-risk cohort.

### 2.1. Patient Selection: Inclusion and Exclusion Criteria

Adult patients (≥18 years) with diabetes mellitus who underwent transmetatarsal, Lisfranc, Chopart, or below-knee amputation for diabetic foot complications and subsequently developed postoperative flap necrosis, wound-healing disorders, or tissue defects requiring further limb-salvage treatment were considered eligible for inclusion. Advanced Wagner or PEDIS stage, osteomyelitis, peripheral ischemia, or unsuccessful revascularization were not considered automatic exclusion criteria, as limb-salvage treatment was pursued whenever clinically feasible. Patients were considered unsuitable for continued limb-salvage treatment when uncontrolled infection, progressive tissue necrosis, proximal extension of osteomyelitis, persistent critical ischemia without a feasible revascularization option, or systemic deterioration made preservation of the existing amputation level clinically unfeasible. Patients were treated at our tertiary referral center. At the time of evaluation, all included patients had a clinical indication for extensive surgical debridement, reamputation, or level-up amputation because of persistent flap necrosis, tissue loss, or non-healing wounds. No predefined postoperative-day interval was required for eligibility; inclusion was based on documented post-amputation tissue failure requiring further limb-salvage treatment. The retrospective clinical records did not permit reliable reconstruction of the intervals from index amputation to recognition of flap failure, from flap failure to treatment allocation, or from treatment allocation to the first iEGF administration for the entire cohort. Therefore, these treatment-timing intervals could not be summarized or incorporated into the comparative analyses.

Patients were excluded if they had incomplete clinical records, were lost to follow-up before outcome assessment, had flap necrosis unrelated to diabetic foot disease (e.g., traumatic), had insufficient clinical data to determine treatment outcomes, or declined the proposed limb-salvage treatment protocol. Patients without postoperative flap necrosis or tissue defects after amputation were not eligible for inclusion. Because screening-level records were not sufficiently standardized to permit reliable reconstruction of the number of patients excluded for each individual criterion, exclusion counts by reason could not be reported retrospectively.

### 2.2. Patient Characteristics and Clinical Data

Demographic, clinical, laboratory, wound-related, treatment-related, and outcome variables were retrospectively collected from the institutional medical records. Demographic and clinical variables included age, sex, smoking status, comorbidities, ASA classification, and type of index amputation. Laboratory parameters recorded at admission/evaluation included fasting blood glucose (mg/dL), hemoglobin (g/dL), white blood cell count (×10^3^/µL), absolute neutrophil count (×10^3^/µL), C-reactive protein (CRP; mg/L), erythrocyte sedimentation rate (mm/h), serum sodium (mEq/L), urea (mg/dL), creatinine (mg/dL), estimated glomerular filtration rate (eGFR; mL/min/1.73 m^2^), and HbA1c (%).

Wound- and disease-related variables included Wagner grade, PEDIS infection grade, presence of osteomyelitis, postoperative flap necrosis, wound-healing impairment or tissue defect, and vascular status based on available angiographic/interventional records. Treatment-related variables included the number of surgical interventions and debridements, revascularization outcome, use of negative pressure wound therapy (NPWT), hyperbaric oxygen therapy (HBOT), split-thickness skin grafting, iEGF treatment, and the total number of iEGF applications. Outcome variables included treatment success, the composite reamputation/level-up amputation outcome, mortality during follow-up, and time to complete wound healing when available. Wagner and PEDIS categories were recorded according to the corresponding established classification systems.

Osteomyelitis was diagnosed based on bone scintigraphy or magnetic resonance imaging (MRI). In patients without available imaging, osteomyelitis was considered present when a positive probe-to-bone test was supported by microbiological confirmation from bone culture.

Decisions regarding reamputation or progression to a more proximal amputation level were based on multidisciplinary clinical assessment rather than on a single absolute criterion. Whenever clinically feasible, preservation of the most distal functional amputation level was prioritized. Reamputation or level-up amputation was considered in the presence of persistent or uncontrolled infection, progressive tissue necrosis, osteomyelitis extending proximal to the existing amputation level, extensive flap necrosis or tissue loss precluding healing at the current level, persistent critical ischemia despite attempted revascularization when feasible, failure of the wound to progress despite serial debridement and multidisciplinary wound care, or systemic deterioration related to ongoing infection. Advanced Wagner or PEDIS stage, osteomyelitis, or peripheral ischemia alone were not considered automatic indications for major amputation; these patients continued to undergo limb-salvage treatment whenever clinically appropriate.

Treatment allocation was not randomized and was based on routine multidisciplinary clinical decision-making. The decision to administer iEGF was individualized and was not determined by a single predefined criterion. Factors considered included the overall clinical condition of the patient, characteristics and suitability of the wound bed, infection control, vascular status and the feasibility or outcome of revascularization, patient acceptance of the proposed treatment, and availability of iEGF during the treatment period. In addition, iEGF became incorporated into the institutional limb-salvage approach during the study period; therefore, temporal differences in treatment availability and clinical practice also influenced treatment allocation. Patients who did not receive iEGF continued to receive conventional multidisciplinary wound and limb-salvage management according to their clinical requirements.

In the study group, intralesional epidermal growth factor (iEGF) was administered according to the institutional treatment protocol. Each treatment session consisted of 75 μg of iEGF prepared in a total volume of 5 mL and divided into five 1 mL syringes. The solution was administered through multiple intralesional and perilesional injections distributed throughout the wound bed and margins. The iEGF dose per treatment session was fixed at 75 μg and was not adjusted according to wound or flap size; the 5 mL solution was distributed across the wound bed and perilesional margins according to the extent of the wound. Treatment was performed three times weekly on alternate days, routinely on Monday, Wednesday, and Friday. No local anesthesia was used. The total number of iEGF applications ranged from 3 to 21 and was determined according to the clinical evolution of the wound and continued suitability for treatment. No treatment-limiting adverse event requiring permanent discontinuation of iEGF was observed. iEGF was administered as part of the multidisciplinary limb-salvage strategy together with other clinically indicated interventions, including serial surgical debridement, revascularization, NPWT, HBOT, and reconstructive procedures when required. The total number of iEGF applications, time to complete wound healing, and requirement for reconstructive procedures were recorded.

The primary endpoint was treatment success, defined as a cumulative clinical status at the last available assessment, consisting of preservation of the original amputation level without reamputation or proximal level-up amputation together with satisfactory wound healing. For the purposes of this analysis, satisfactory wound healing was defined as a viable and progressively healing wound without progressive tissue necrosis, uncontrolled local infection, or a clinical indication for further reamputation or proximal level-up amputation. Complete epithelialization was not required for this component of the composite endpoint. This endpoint therefore represents a cumulative clinical status at the last available assessment rather than a standardized time-point measure of complete wound healing. Reamputation and level-up amputation were recorded as a single composite amputation-related outcome and were not analyzed as separate endpoints. Secondary endpoints included mortality and, in the flow cytometry sub-study, changes in the proportions of CD38^+^ and CD209^+^ cell proportions within the CD45^+^CD14^+^ macrophage-enriched population between pre- and post-treatment wound tissue samples. All clinical outcomes were assessed over the entire available follow-up period from study inclusion to the last documented clinical assessment or death, with follow-up censored in December 2025. Mortality was defined as death from any cause documented during the available follow-up period. The median follow-up duration for the entire cohort was 410 days (range, 13–2435 days).

### 2.3. Cell Isolation and Immunophenotyping

The flow cytometry sub-study included 12 iEGF-treated patients for whom paired wound tissue samples obtained before and after treatment were available. Pre-treatment biopsies were harvested from the active granulation tissue bed at the amputation site during initial debridement before the first iEGF application. Post-treatment biopsies were obtained from comparable viable granulation regions of the same wound bed upon completion of the planned iEGF treatment cycle. The exact number of days between the paired biopsies was not consistently recorded in the retrospective clinical records; therefore, the sampling interval was defined clinically as the period from the initial pre-treatment debridement to completion of the planned iEGF treatment cycle, after a median of 8 applications (range: 3–21). Macroscopically visible necrotic areas were carefully removed under laminar flow conditions, and viable granulation tissues were divided into 1–2 mm^3^ fragments. Tissue processing and flow cytometric acquisitions were standardized across all samples according to the same institutional protocol; cytometric analyses were performed in an unblinded manner as part of the clinical substudy workflow. To obtain a single-cell suspension, samples were enzymatically digested for 45 min at 37 °C with continuous agitation using collagenase type I (1 mg/mL; Sigma-Aldrich, St. Louis, MO, USA) in RPMI-1640 medium. The resulting cell suspension was filtered through a 40 µm cell strainer (BD Biosciences, San Jose, CA, USA) and washed twice with sterile phosphate-buffered saline (PBS) containing 2% fetal bovine serum. Cells were stained with fluorescently conjugated monoclonal antibodies against human CD45-Pacific Blue (clone 2D1, cat. no. 368539), CD14-APC-Cy7 (clone 63D3, cat. no. 367107), CD38-FITC (clone HB-7, cat. no. 356609), and CD209-PE (clone DCS-8C1, cat. no. 343005) (BioLegend, San Diego, CA, USA). Propidium iodide was used as a viability dye (cat. no. P4170; Sigma-Aldrich, Taufkirchen, Germany). A macrophage-enriched population was operationally defined as CD45^+^CD14^+^ events. The gating strategy consisted of sequential exclusion of debris using FSC-A/SSC-A characteristics, exclusion of doublets using FSC-A versus FSC-H, and selection of viable cells based on propidium iodide negativity. Within the viable single-cell population, leukocytes were identified as CD45^+^ events, followed by selection of CD14^+^ cells. CD38^+^ and CD209^+^ expression was then evaluated within the CD45^+^CD14^+^ macrophage-enriched population using a FACSCanto II flow cytometer and FACSDiva software (v8.0.1, BD Biosciences, San Jose, CA, USA). The complete gating strategy is shown in Supplementary Figure S1.

### 2.4. Statistical Analysis

Statistical analyses were performed using the software Jamovi (version 2.7.37, The jamovi project, Sydney, Australia), and all tests were two-sided with a predefined significance level of *p* < 0.05. Data were expressed as number (*n*) and percentage (%), mean ± standard deviation, or median (minimum–maximum), as appropriate. Normality of continuous variables was assessed using the Kolmogorov–Smirnov test. Missing data were not imputed. Except for vascular status, analyses were performed using available observations for each variable. Analyses incorporating vascular status were restricted to patients with available angiographic/interventional data and therefore represented complete-case analyses for this variable.

Changes in the paired percentages of CD38^+^ and CD209^+^ cells within the CD45^+^CD14^+^ macrophage-enriched population were assessed using the paired Student’s t-test for normally distributed data or the Wilcoxon signed-rank test when the normality assumption was not met.

No formal a priori sample-size calculation was performed because of the retrospective nature of the study. Given the small number of treatment failures in the iEGF group, the multivariable analysis was intentionally restricted to a parsimonious model, and Firth penalized logistic regression was used as a sensitivity analysis to reduce sparse-data bias and model instability.

Univariate analyses were performed using the chi-square test for categorical variables and Student’s t-test or the Mann–Whitney U test for continuous variables, depending on data distribution. The Spearman correlation test was used to evaluate linear relationships between continuous variables.

To identify factors associated with treatment success after adjustment for selected measured covariates, multivariable logistic regression analysis was performed. A multivariable model incorporating iEGF treatment, age, HbA1c, and vascular status was performed as a sensitivity analysis to examine whether the observed association persisted after conditioning on these selected measured clinical variables. Vascular status was included because tissue perfusion is clinically relevant to wound healing and limb-salvage outcomes; however, because the temporal relationship between revascularization and iEGF initiation could not be reliably reconstructed, vascular/interventional status may partly reflect the evolving treatment pathway rather than a purely baseline confounder. Although univariate associations were examined, the final covariate set was not determined solely by statistical significance. The model was intentionally kept parsimonious to reduce the risk of overfitting and because several potentially relevant variables were unavailable, incompletely recorded, or occurred during follow-up rather than at baseline. Effect estimates were reported as adjusted odds ratios (aORs) with 95% confidence intervals (CIs). Given the non-randomized treatment allocation and the limitations of the retrospective dataset, adjusted estimates were interpreted as associations conditional on the measured covariates included in the model rather than as estimates of an independent causal treatment effect. Adjunctive therapies administered during follow-up, including NPWT, HBOT, and grafting, were not treated as baseline covariates in the multivariable sensitivity model because their timing was not standardized and their use could represent time-dependent mediators or consequences of the evolving clinical course.

Because the number of treatment failures among patients receiving EGF was small, raising concern for sparse-data bias and model instability, a Firth penalized logistic regression analysis was additionally performed as a sensitivity analysis. The consistency of the findings between the conventional multivariable and Firth penalized sensitivity models was used to assess the robustness of the observed associations.

Additionally, the infection outcome showed complete separation from treatment success and could not be estimated using standard logistic regression; therefore, it was reported descriptively. Model fit was evaluated using Akaike’s Information Criterion (AIC), McFadden’s pseudo-R^2^, and likelihood ratio statistics. These statistics were used solely to characterize model fit and were not interpreted as evidence of causal validity. Collinearity was assessed using variance inflation factors (VIFs).

A secondary exploratory analysis was conducted in the subgroup of patients who received EGF (EGF = 1) to examine associations between selected clinical variables and treatment success within this population. Because only four treatment failures occurred in this subgroup, the multivariable model was constructed parsimoniously according to events-per-variable considerations. Osteomyelitis was excluded from multivariable modelling because of complete separation resulting from zero cells in one outcome category and was assessed using Fisher’s exact test. In this subgroup, the LRINEC score was examined exploratorily for its association with treatment success. LRINEC was examined exploratorily as an available composite laboratory measure of systemic inflammatory and metabolic abnormalities and was not considered a validated prognostic instrument for post-amputation flap necrosis. Neutrophil count was additionally examined in sensitivity analyses but was not retained in the final exploratory model because it did not contribute significantly to the model. A two-sided *p* value < 0.05 was considered statistically significant.

## 3. Results

A total of 164 patients were included in this study. Of these, 75.6% (*n* = 124) were male, and 24.4% (*n* = 40) were female, with a median age of 63 years (range: 23–91). The median follow-up duration for the entire cohort was 410 days (range, 13–2435 days). Baseline biochemical parameters and wound characteristics are presented in Table 1 and Table 2, respectively.

Osteomyelitis was detected in 36.6% (*n* = 60) of patients. At diagnosis, the majority of patients had PEDIS stage 3–4 infection, indicating a progressive clinical course with a high risk of limb loss. Only 30 patients (18.3%) had PEDIS stage 2 infection.

Among patients presenting with active infection, infection was controlled during follow-up in 47.6% (*n* = 78), remained uncontrolled in 31.7% (*n* = 52), and was already under control at presentation in 20.7% (*n* = 34).

Regarding the vascular status of the affected extremities, 21.3% of patients (*n* = 35) did not undergo angiography. Successful revascularization was achieved in 42.1% (*n* = 69), partial revascularization in 20.7% (*n* = 34), and no revascularization in 15.9% of patients (*n* = 26).

**Figure 1:**
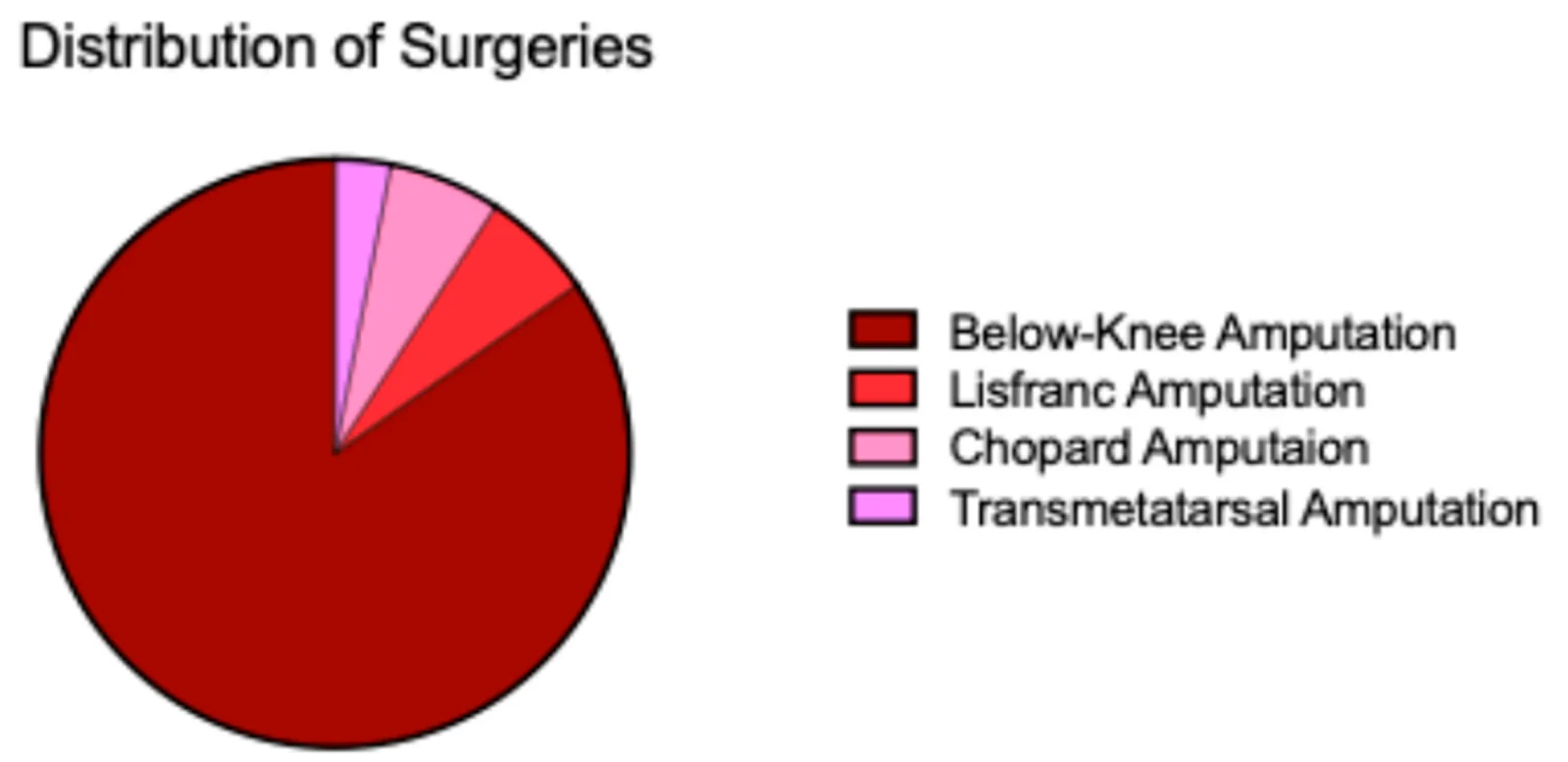
The distribution of surgeries.

During follow-up, 56.7% (*n* = 93) of patients received negative pressure wound therapy (NPWT), 39% (*n* = 64) received hyperbaric oxygen therapy (HBOT), and 15.2% (*n* = 25) underwent split-thickness skin grafting. Intralesional EGF was administered in 51.2% (*n* = 84) of patients, with a median of eight applications (range: 3–21). The distribution of surgical procedures is shown in Figure 1. The median number of surgical interventions during the treatment period was 2 (range: 1–10).

Overall, the study cohort represented a particularly high-risk population. More than 90% of patients presented with Wagner stage 4 disease, nearly 80% had PEDIS stage 3 infection, and over one-third had osteomyelitis. Despite this advanced disease profile, overall treatment success was achieved in 68.3% of patients, whereas the observed crude cumulative mortality proportion was 21.3% (*n* = 35).

At the end of follow-up, overall treatment was considered successful in 68.3% (*n* = 112) and unsuccessful in 31.7% of patients (*n* = 52). The observed crude cumulative mortality proportion was 21.3% (*n* = 35). When outcomes were descriptively stratified according to the initial partial-foot amputation level, treatment failure rates were 20% following Lisfranc amputation, 28% following modified Chopart amputation, and 46% following conventional Chopart amputation. These subgroup findings were considered descriptive because of the limited and heterogeneous sample sizes within individual amputation categories.

NPWT, HBOT, and graft surgery were significantly associated with treatment success (*p* < 0.001, *p* = 0.030, and *p* = 0.021, respectively). The total number of surgical interventions during treatment was also significantly associated with outcome (*p* < 0.001). A weak but statistically significant positive correlation was observed between HbA1c and the LRINEC score (*p* < 0.001, r = 0.297).

Of the total cohort, 84 patients (51.2%) received EGF (study group), while 80 patients (48.8%) did not (control group). The two groups were compared in terms of demographic features, the ASA score, the LRINEC score, biochemical parameters, the presence of osteomyelitis, the Wagner stage, the PEDIS stage, and the total number of surgical procedures. Although several baseline characteristics were similar between groups, clinically relevant imbalances were present. Patients in the iEGF group were younger (*p* = 0.025), while their WBC (*p* = 0.038), neutrophil count (*p* = 0.029), fasting blood glucose (*p* = 0.046), and HbA1c levels (*p* = 0.005) were higher than those of the control group. In addition, vascular status and the use of adjunctive therapies differed substantially between groups, as detailed below.

Additional clinical characteristics and adjunctive treatments are summarized in Table 3. The prevalence of osteomyelitis was comparable between the iEGF and control groups (33.3% vs. 40.0%, respectively; *p* = 0.376). Vascular/interventional data were available for 129 of 164 patients. Vascular data were unavailable for 13 of 84 patients in the iEGF group (15.5%) and 22 of 80 patients in the control group (27.5%); therefore, analyses incorporating vascular status were restricted to these 129 complete cases. Among these patients, vascular status differed significantly between groups (*p* < 0.001), with successful vascular patency more frequently achieved in the iEGF group. Graft surgery, HBOT, and NPWT were also more frequently used in the iEGF group than in the control group (25.0% vs. 5.0%, *p* < 0.001; 48.8% vs. 28.7%, *p* = 0.008; and 71.4% vs. 41.3%, *p* < 0.001, respectively).

**Figure 2:**
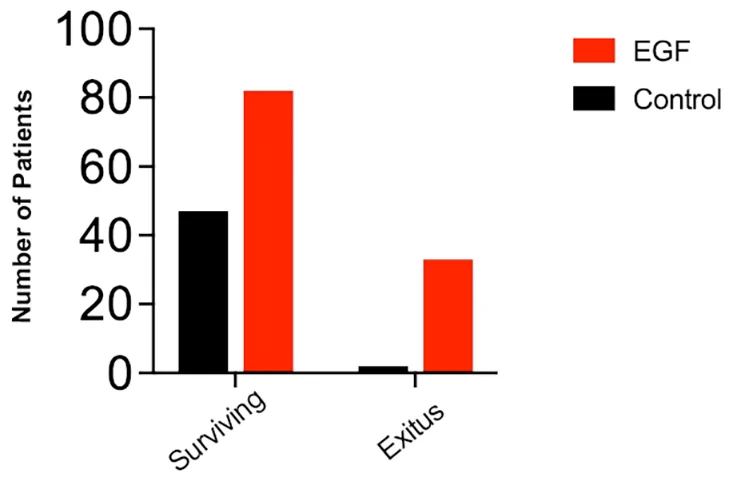
The comparison of mortality between the EGF group and the control group.

The success rate was significantly higher in the iEGF group (95.2% [80/84] vs. 40.0% [32/80], *p* < 0.001). The observed crude cumulative mortality proportion was lower in the iEGF group (2.4% [2/84] vs. 41.3% [33/80], *p* < 0.001) (Figure 2). Among successfully treated patients in the iEGF group, the number of EGF doses administered was significantly higher (*p* < 0.001).

As a sensitivity analysis, a multivariable logistic regression model incorporating iEGF treatment, age, HbA1c, and vascular status was performed among patients with available vascular information. In this model, iEGF treatment remained associated with favorable outcome (aOR = 39.43, 95% CI 8.17–190.57, *p* < 0.001). Increasing age was associated with lower odds of treatment success (aOR = 0.94, *p* = 0.040), whereas HbA1c was not associated with outcome (*p* = 0.991). Compared with patients who achieved successful revascularization, patients with unsuccessful revascularization had significantly lower odds of treatment success (aOR = 0.19, *p* = 0.021).

To assess the potential impact of sparse-data bias resulting from the small number of failures among EGF-treated patients, a Firth penalized logistic regression analysis was performed as a sensitivity analysis. The findings were directionally consistent with the conventional multivariable sensitivity model. iEGF treatment remained associated with favorable outcome (aOR = 27.52, 95% CI 7.86–146.85, *p* < 0.001). The wide confidence intervals surrounding the estimates from both models indicate substantial uncertainty regarding the magnitude of the observed association. Accordingly, these adjusted odds ratios should be interpreted as measures of association conditional on the included covariates rather than as estimates of an independent treatment effect. Increasing age (aOR = 0.94, 95% CI 0.89–1.00, *p* = 0.039) and unsuccessful revascularization (aOR = 0.22, 95% CI 0.05–0.80, *p* = 0.021) were associated with lower odds of treatment success, whereas HbA1c and partially successful revascularization were not significantly associated with outcome. Detailed effect estimates from both models are presented in Table 4.

Among patients who received EGF, an exploratory association was observed between the LRINEC score and treatment success (aOR per one-point increase = 0.27, 95% CI = 0.10–0.70, *p* = 0.007). Given the occurrence of only four treatment failures in this subgroup, this estimate should be considered unstable and hypothesis-generating rather than evidence of prognostic validity.

**Figure 3:**
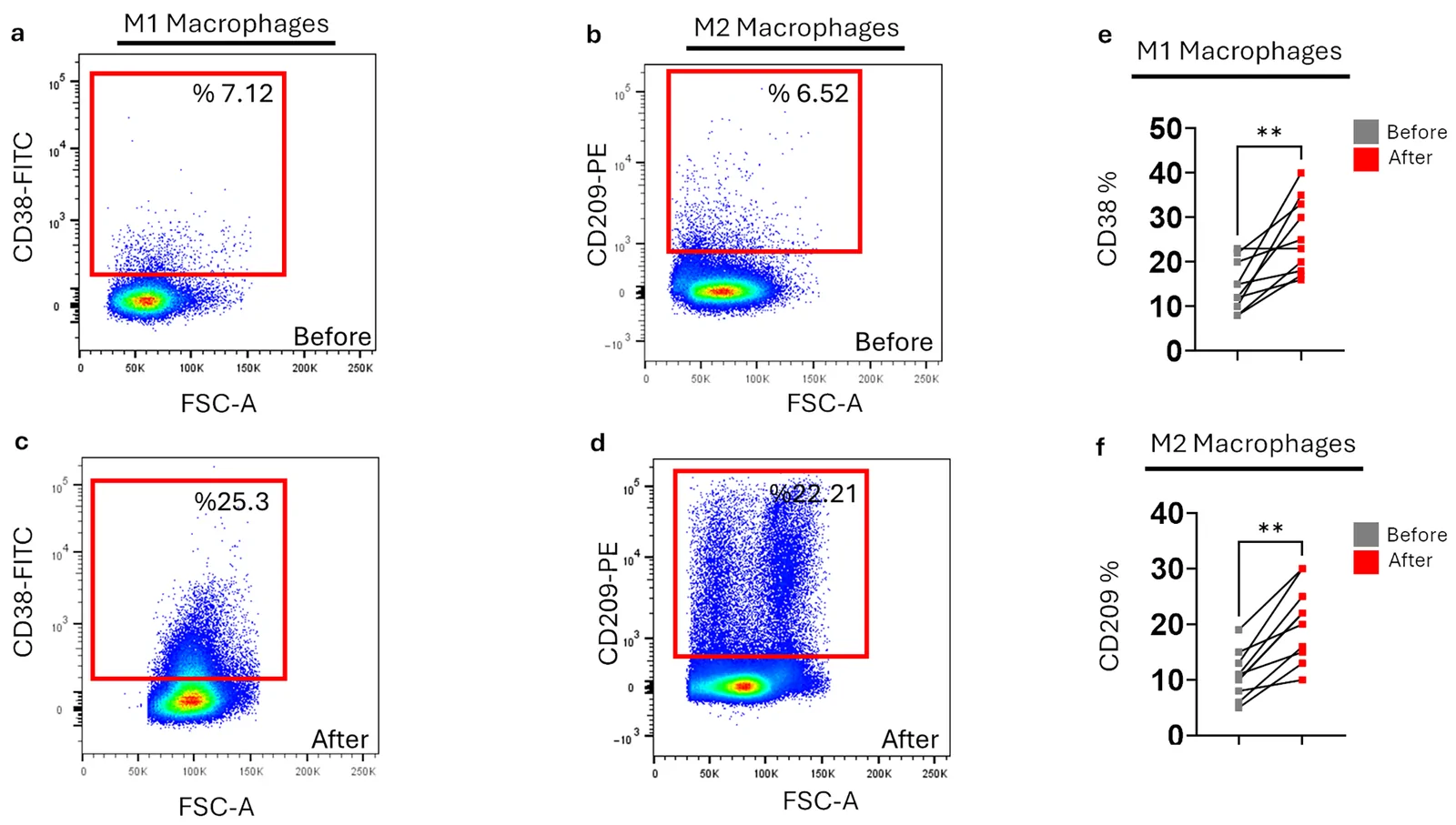
Representative flow cytometry plots and paired pre–post changes in CD38^+^ and CD209^+^ cell proportions within the CD45^+^CD14^+^ macrophage-enriched population following the iEGF treatment cycle. (**a**) Pre-treatment CD38^+^ cell population; (**b**) pre-treatment CD209^+^ cell population; (**c**) post-treatment CD38^+^ cell population; (**d**) post-treatment CD209^+^ cell population; (**e**) paired pre–post changes in CD38^+^ cell proportions; (**f**) paired pre–post changes in CD209^+^ cell proportions. Red boxes indicate the gated positive cell populations. ** indicates *p* < 0.001.

Flow-cytometric analysis of paired wound-tissue samples obtained before and after treatment demonstrated an increase in the proportions of both CD38^+^ and CD209^+^ cells within the CD45^+^CD14^+^ macrophage-enriched population in the iEGF-treated subgroup (*n* = 12). The mean proportion of CD38^+^ cells increased from 10.8% before treatment to 20.3% after treatment, corresponding to a mean difference of 9.5 percentage points (95% CI: 4.1–14.82; *p* < 0.001). Similarly, the proportion of CD209^+^ cells increased from 14.5% to 25.7%, corresponding to a mean difference of 11.2 percentage points (95% CI: 4.52–17.8; *p* < 0.001; Figure 3). These pre–post changes were exploratory and do not establish macrophage polarization or a causal effect of iEGF.

## 4. Discussion

The primary objective in the management of diabetic foot (DF) is to avoid major amputation and preserve a functional limb whenever possible. Major amputation is associated with increased morbidity and mortality, reduced prosthesis utilization, and marked limitation in mobility [3,19,20]. Loss of mobility further disrupts metabolic balance and increases cardiovascular complications, negatively affecting long-term survival. In our cohort, iEGF administered as part of multidisciplinary wound care was associated with higher treatment success, while a lower crude cumulative mortality proportion was observed in the iEGF group compared with the control group. Given the magnitude of the observed mortality difference and the non-randomized treatment-selection process, this finding cannot be attributed to iEGF alone and may reflect differences in baseline prognosis, vascular status, access to multidisciplinary care, treatment timing, and survival to treatment. Accordingly, the mortality finding should be regarded as a crude observational comparison rather than evidence of a treatment-associated survival benefit.

Reamputation remains a frequent complication following DF surgery. Previous reports have identified multiple ulcers, peripheral artery disease, elevated CRP levels, peripheral neuropathy, and the absence of distal pulses as important risk factors [13]. The coexistence of these factors reduces the probability of wound healing at a given amputation level and often necessitates additional surgical intervention. The observed variation in treatment failure across amputation levels further highlights the clinical heterogeneity of this post-amputation population and the importance of careful patient selection, vascular optimization, and infection control in limb-salvage strategies.

Amputations in diabetic patients are known to carry a higher complication risk compared with non-diabetic populations, including flap necrosis, wound dehiscence, prolonged hospitalization, and increased reamputation rates [21]. Several clinically relevant differences were present between the treatment groups. Patients receiving iEGF were younger but had higher WBC and neutrophil counts, fasting blood glucose, and HbA1c levels. In addition, among patients with available angiographic/interventional data, successful vascular patency was more frequently achieved in the iEGF group. These imbalances indicate that the two groups were not fully comparable and should be considered when interpreting the observed treatment differences. In the adjusted analysis, iEGF treatment remained associated with treatment success, whereas increasing age and unsuccessful revascularization were associated with lower odds of success; HbA1c was not associated with outcome [22]. The direction of these findings remained consistent in the Firth penalized sensitivity analysis. Nevertheless, because of the retrospective and non-randomized design, these adjusted associations should not be interpreted as evidence of a causal treatment effect. Furthermore, the magnitude of the observed association should not be interpreted as the magnitude of an effect attributable to iEGF itself, because residual confounding and differences in the overall multidisciplinary limb-salvage strategy cannot be excluded.

Importantly, the magnitude of the observed association in the present cohort was substantially greater than the treatment effects generally reported in randomized studies and meta-analyses of EGF therapy for conventional diabetic foot ulcers. The 55.2-percentage-point absolute difference in treatment success between the iEGF and control groups should therefore not be interpreted as an estimate of the causal treatment effect of iEGF. Our cohort represents a highly selected post-amputation population at immediate risk of reamputation or proximal level-up amputation, and iEGF was administered within a broader multidisciplinary limb-salvage pathway. Differences in patient selection, treatment timing and opportunity, vascular optimization, adjunctive therapies, and other measured or unmeasured prognostic factors may all have contributed to the magnitude of the observed association. Accordingly, the present findings should be regarded as hypothesis-generating rather than as evidence of the magnitude of an independent iEGF treatment effect.

The observed positive correlation between HbA1c and the LRINEC score further suggests that glycemic control is closely related to infection severity. Diabetes is a well-established comorbidity in necrotizing soft tissue infections and has been associated with higher amputation rates [23,24,25]. Our findings are consistent with the existing literature indicating that poor glycemic regulation contributes to a more severe infectious course [26,27]. Given that LRINEC was originally developed for necrotizing soft-tissue infection and only four treatment failures occurred in the iEGF subgroup, its association with outcome in the present post-amputation cohort should be regarded as exploratory and should not be interpreted as evidence of validated prognostic performance. Because this analysis was not intended to develop or validate a prediction model, and because of the very small number of outcome events, discrimination measures and internal validation were not considered reliable or appropriate.

Proper wound bed preparation remains a cornerstone of DF management. Repeated and meticulous debridement removes necrotic tissue and biofilm, facilitating the transition from a chronic inflammatory environment to a more active healing phase. In our study, the association between the number of surgical interventions and treatment success underscores the importance of structured surgical management. This finding also underscores that the observed association between biological adjuncts such as iEGF and treatment success should be interpreted within the context of adequate wound-bed preparation and comprehensive multidisciplinary care [28]. Regenerative approaches are particularly relevant in patients with extensive tissue defects following minor amputations. iEGF has been shown to enhance fibroblast and epithelial cell proliferation while supporting angiogenesis and extracellular matrix remodeling [29,30,31]. Experimental studies have also demonstrated enhanced wound healing following topical EGF administration [32]. The higher number of iEGF doses observed among successfully treated patients should be interpreted cautiously, as this association may reflect longer treatment opportunity, reverse causation, or treatment-duration bias rather than a biological dose–response effect. Accordingly, no formal dose–response analysis was performed, because the number of applications represented a time-dependent exposure influenced by the evolving clinical trajectory and could not be appropriately evaluated without time-dependent methodology.

In addition to adequate debridement, current IWGDF recommendations emphasize that optimal outcomes in diabetic foot management depend on effective infection control, restoration of tissue perfusion through timely revascularization, and appropriate offloading strategies [5]. Adjunctive modalities such as NPWT and HBOT have also been investigated for their effects on wound healing and tissue regeneration and were frequently used in our cohort; however, these core principles remain fundamental [33,34,35]. In this context, the observed association between iEGF use and treatment success should be interpreted within a comprehensive treatment approach that includes a well-prepared wound bed, controlled infection, sufficient perfusion, and reduced mechanical stress.

Current international guidelines, including the 2023 IWGDF wound-healing recommendations, do not support the routine use of growth-factor therapies in diabetic foot ulcers because of the low certainty of the available evidence and the limited evidence regarding patient-important outcomes such as sustained healing, amputation, quality of life, resource utilization, and mortality [5]. Therefore, the present observational findings should not be interpreted as challenging or modifying current guideline recommendations. However, our cohort represents a more selected group of patients with post-amputation flap necrosis and tissue defects—clinical situations that are relatively underrepresented in conventional DFU studies and often occur at a critical stage between limb salvage and progression to more proximal amputation. For this reason, our findings should be interpreted within this specific clinical setting rather than generalized to routine DFU management. Accordingly, the present findings should be viewed as an extension of the existing clinical hypothesis regarding iEGF to this distinct post-amputation population, rather than as evidence that current guideline recommendations should be revised.

Chronic diabetic wounds are characterized by persistent inflammation, resulting in impaired macrophage plasticity, continued production of pro-inflammatory mediator factors like IL-1, IL-6, and TNF-α, defective efferocytosis, and delayed transition toward reparative (anti-inflammatory) phenotypes, all of which contribute to impaired tissue regeneration. CD38 has been associated with inflammatory macrophage activation and cellular metabolic regulation, whereas CD209 is commonly expressed by macrophages involved in antigen recognition and tissue remodeling. Nevertheless, neither marker alone is sufficient to define macrophage functional identity. The flow-cytometry substudy demonstrated an increase in CD38- and CD209-expressing cells within the CD45^+^CD14^+^ macrophage-enriched population. These findings may indicate changes in the cellular composition or activation state of the wound microenvironment. Rather than representing stable terminal phenotypes, macrophages exhibit phenotypic plasticity in response to local microenvironmental factors, including hypoxia, inflammatory cytokines, growth factors, extracellular matrix remodeling, and metabolic signals. Consequently, the expression of individual surface markers reflects activation states rather than discrete macrophage subsets with their terminal differentiation. However, macrophage phenotypes exist along a dynamic functional continuum and cannot be fully characterized using a binary M1/M2 framework. Accordingly, CD38 and CD209 were interpreted as inflammatory- and reparative-associated markers, respectively, within the CD45^+^CD14^+^ macrophage-enriched population, rather than as definitive or mutually exclusive indicators of M1 and M2 macrophage phenotypes. As the analysis was limited to two surface markers, the observed changes should be considered preliminary and hypothesis-generating. Because this substudy was not designed to assess mediation and included only 12 patients, no formal analysis was performed linking the magnitude of CD38/CD209 changes with clinical outcomes. Therefore, the observed pre–post changes cannot establish that iEGF induced macrophage polarization or that these cellular changes mediated the association between iEGF exposure and limb-salvage outcomes. Future studies incorporating broader marker panels, including CD163, CD206, and HLA-DR, together with functional or transcriptomic analyses, are needed to more comprehensively characterize macrophage responses to iEGF. No mechanistic experiments were performed to establish how iEGF produced the observed clinical or cellular changes; therefore, the present findings should be interpreted within the context of an observational study, and the flow-cytometry substudy should be regarded as exploratory rather than mechanistic.

As shown in Table 3, the more frequent use of adjunctive therapies, including NPWT, HBOT, and graft surgery, together with differences in vascular management, indicates that iEGF was commonly administered as part of a broader and substantially different multidisciplinary limb-salvage strategy rather than as an isolated intervention. These treatment imbalances are important because the individual contribution of each modality cannot be reliably separated in this observational cohort. Moreover, these adjunctive therapies were applied during follow-up and may partly reflect clinical evolution and the opportunity to continue limb-salvage treatment, rather than baseline treatment assignment alone. Patients with rapidly progressive disease or early need for major surgery may have had less opportunity to receive prolonged adjunctive therapies. Therefore, the observed differences in outcomes should be interpreted as arising within a multimodal treatment context, and the independent contribution of iEGF cannot be fully isolated.

Despite advances in conventional wound care, healing rates for chronic wounds remain limited, with reported rates of 24–30% within 12–20 weeks [35]. Therefore, the integration of regenerative therapies into standard treatment algorithms may provide additional benefit, particularly in patients who develop complications after minor amputations [16,36,37,38].

This study has several limitations. First, its observational, non-randomized design, together with retrospective identification and data collection, introduces the potential for selection bias and confounding by indication. Treatment allocation was based on individualized multidisciplinary clinical decision-making and was influenced by clinical condition, wound characteristics, infection and vascular status, patient acceptance, treatment availability, and temporal changes in institutional practice. Although these factors reflect real-world clinical care, they limit direct causal interpretation of the observed association between iEGF treatment and clinical outcomes. In addition, iEGF and several adjunctive treatments were initiated during follow-up rather than assigned at a common baseline. Consequently, patients who remained clinically stable and eligible for continued limb-salvage treatment may have had greater opportunity to receive these interventions than patients who experienced early treatment failure, introducing the possibility of time-dependent treatment-selection and immortal-time bias. The retrospective dataset did not permit reliable reconstruction of the complete temporal sequence of treatment initiation for all patients, and this potential bias could not be formally addressed in the present analysis. This limitation is fundamental to the primary comparison and precludes interpretation of the observed association as an estimate of the causal treatment effect of iEGF.

Second, important treatment imbalances existed between the groups. NPWT, HBOT, graft surgery, and successful vascular intervention were more frequent in the iEGF group. These modalities formed part of a broader multidisciplinary limb-salvage strategy and their individual effects cannot be reliably separated from that of iEGF in this retrospective cohort. Furthermore, because NPWT, HBOT, and grafting were administered during follow-up rather than assigned at baseline, their use may also have been influenced by clinical evolution and the opportunity to continue limb-salvage treatment. Although vascular status was included in the adjusted model, residual confounding related to revascularization and other co-interventions remains possible. In addition, vascular-adjusted analyses were limited to the 129 patients with available angiographic/interventional data. Because the reasons for absence of angiographic assessment were not consistently documented, the possibility of selection bias related to complete-case analysis cannot be excluded. Furthermore, the exact temporal relationship between revascularization and iEGF initiation could not be reliably reconstructed; therefore, vascular/interventional status may partly reflect the evolving treatment pathway rather than a purely baseline confounder.

Third, the number of treatment failures in the iEGF group was small (*n* = 4), limiting the number of covariates that could be reliably included in multivariable analysis and increasing the risk of sparse-data bias and model instability. To mitigate this limitation, the conventional multivariable sensitivity model was intentionally kept parsimonious, and Firth penalized logistic regression was performed as an additional sensitivity analysis, yielding directionally consistent findings. Nevertheless, the large effect estimates and their uncertainty should be interpreted cautiously and should not be regarded as definitive evidence of a causal treatment effect.

Fourth, several potentially relevant variables, including diabetes duration, nutritional status, previous ulcer history, duration of infection before referral, detailed antibiotic treatment protocols, and surgeon-specific treatment decisions, were not consistently available in the retrospective dataset and could not be incorporated into the analyses. Detailed classification of wounds as neuropathic, ischemic, or neuroischemic and subclassification of infection phenotypes were also not available. Propensity score matching or inverse probability weighting was not performed. Given the limited sample size, sparse treatment failures in the iEGF group, incomplete vascular data, and time-dependent co-interventions, a post hoc propensity analysis was not considered sufficient to resolve the principal confounding concerns; moreover, such methods would not address unmeasured confounding.

Finally, the flow cytometry analysis was performed in a small subset of iEGF-treated patients with available paired tissue samples, and macrophage characterization was limited to CD38 and CD209 expression. This substudy did not include an untreated group with paired tissue sampling; therefore, the observed pre–post cellular changes cannot distinguish an iEGF-specific effect from the immunological evolution of wound healing or the effects of concomitant interventions. In addition, reamputation and proximal level-up amputation were recorded as a composite endpoint and could not be reliably analyzed separately, limiting the ability to distinguish between outcomes with different functional consequences. The primary endpoint was assessed at the last available clinical follow-up rather than at a standardized prespecified time point. Because follow-up duration was heterogeneous and reliable dates for complete epithelialization, reamputation, and proximal level-up amputation could not be consistently reconstructed from the retrospective records, time-to-event analyses and standardized complete-healing outcomes could not be performed. Accordingly, the primary endpoint should be interpreted as a cumulative status at last assessment rather than as a standardized measure of wound-healing kinetics. Because group-specific follow-up distributions and reliable time-to-event data could not be reconstructed from the retrospective dataset, the observed mortality difference should be interpreted as a crude cumulative comparison rather than as a time-adjusted treatment effect. Long-term functional outcomes and quality-of-life measures were not systematically assessed. Accordingly, the clinical and immunological findings should be considered as exploratory and hypothesis-generating and require confirmation in prospective, adequately powered multicenter studies with standardized treatment protocols and more comprehensive immunophenotypic assessment.

## 5. Conclusions

In this observational cohort of high-risk diabetic foot patients with post-amputation flap necrosis, wound-healing disorders, or tissue defects, iEGF administered as part of a multidisciplinary limb-salvage strategy was associated with higher treatment success, while a lower crude cumulative mortality proportion was also observed in the iEGF group; this mortality difference should not be interpreted as evidence of a treatment-associated survival benefit. These findings suggest that iEGF may represent a potentially useful adjunct within a comprehensive limb-salvage approach in selected high-risk patients; however, its individual contribution cannot be separated from the effects of vascular optimization, infection control, surgical debridement, and other adjunctive therapies. The non-randomized treatment allocation, treatment imbalances, heterogeneous follow-up, and residual confounding preclude causal interpretation, and the observed associations should therefore be regarded as hypothesis-generating and requiring prospective confirmation rather than as evidence of an independent treatment effect. The pre–post changes in the proportions of CD38^+^ and CD209^+^ cells within the CD45^+^CD14^+^ macrophage-enriched population provide exploratory evidence of alterations in the wound immune microenvironment but do not establish macrophage polarization or a causal biological mechanism. Prospective controlled multicenter studies with standardized treatment protocols, predefined treatment timing and follow-up, time-to-event outcomes, and comprehensive immunophenotyping are required to determine the clinical contribution of iEGF in this specific post-amputation population.

## Figures and Tables

**Table 1:** The comparison of biochemical parameters at admission between the EGF group and the control group.

Biochemical Parameters	Overall	Groups		*p*-Value
		EGF Group	Control Group	
Fasting Blood Glucose (mg/dL)	205 (60–862)	234 (63–862)	190 (60–619)	0.046 *
Hemoglobin (g/dL)	10.9 ± 1.84	11.1 ± 1.814	10.7 ± 1.86	0.199
WBC (×10^3^/uL)	13.1 (4.6–41.6)	14.8 (4.6–40.2)	11.1 (5.0–41.6)	0.038 *
Neutrophil count (×10^3^/µL)	9.9 (2.5–35.6)	11.9 (2.6–35.3)	8.75 (2.5–35.6)	0.029 *
CRP (mg/L)	122 (2–473)	149 (2.5–425)	106 (2–473)	0.063
Sedimentation Rate (mm/h)	83.2 ± 29.4	84.9 ± 30.2	81.3 ± 28.7	0.436
Na (mEq/L)	135 (121–143)	134 (126–142)	136 (121–143)	0.265
Urea (mg/dL)	46 (12–190)	45.5 (16–168)	47.5 (15–190)	0.322
Creatinine (mg/dL)	1.10 (0.4–7.87)	1.12 (0.47–7.87)	1.04 (0.40–7.70)	0.543
eGFR (mL/min/1.73 m^2^)	67.5 (7–150)	66 (8–150)	70.5 (7–150)	0.833
HbA1c (%)	9.5 (5.3–15.8)	9.8 (6.3–15)	8.9 (5.3–15)	0.005 *

(Data were expressed as mean ± standard deviation if the data were normally distributed, or median (minimum–maximum) if the data were not normally distributed). * *p* < 0.05 was considered statistically significant.

**Table 2:** The Wagner and PEDIS classifications at admission.

Classification System	Overall	Groups		*p*-Value
		EGF Group	Control Group	
**Wagner**				
• Stage 1	0	0	0	0.204
• Stage 2	0	0	0	
• Stage 3	10 (6.1%)	6 (7.1%)	4 (5%)	
• Stage 4	151 (92.1%)	78 (92.9%)	73 (91.3%)	
• Stage 5	3 (1.8%)	0	3 (3.8%)	
**PEDIS**				
• Stage 1	0	0	0	0.255
• Stage 2	30 (18.3%)	19 (22.6%)	11 (13.8%)	
• Stage 3	130 (79.3%)	64 (76.2%)	66 (82.5%)	
• Stage 4	4 (2.4%)	1 (1.2%)	3 (3.8%)	

(Data were expressed as *n* (column %)).

**Table 3:** Osteomyelitis, vascular status, and adjunctive treatments according to treatment group (Data were expressed as *n* (column %)).

	Overall	Groups		*p*-Value
		EGF Group	Control Group	
Osteomyelitis				
• Yes	60 (36.6%)	28 (33.3%)	32 (40%)	0.376
• No	104 (63.4%)	56 (66.7%)	48 (60%)	
Vascular Status				
• Unsuccessful intervention	26 (20.2%)	7 (9.9%)	19 (32.8%)	<0.001 *
• Partially successful intervention	34 (26.4%)	13 (18.3%)	21 (36.2%)	
• Vascular patency achieved with intervention	69 (53.5%)	51 (71.8%)	18 (31%)	
Graft Surgery				
• Yes	25 (15.2%)	21 (25%)	4 (5%)	<0.001 *
• No	139 (84.8%)	63 (75%)	76 (95%)	
HBOT				
• Yes	64 (39%)	41 (48.8%)	23 (28.7%)	0.008 *
• No	100 (61%)	43 (51.2%)	57 (71.3%)	
NPWT				
• Yes	93 (56.7%)	60 (71.4%)	33 (41.3%)	<0.001
• No	71 (43.3%)	24 (28.6%)	47 (58.8%)	

Note: Vascular status percentages were calculated among patients with available angiographic/interventional data (*n* = 129; EGF group, *n* = 71; control group, *n* = 58). Thirty-five patients did not undergo angiography. Accordingly, angiographic/interventional data were unavailable for 13 of 84 patients (15.5%) in the iEGF group and 22 of 80 patients (27.5%) in the control group. Percentages for the remaining variables were calculated using the total number of patients in each treatment group. * *p* < 0.05 was considered statistically significant.

**Table 4:** Sensitivity analyses of factors associated with treatment success among patients with available vascular data.

Variable	Conventional Multivariable Logistic Regression aOR (95% CI)	*p*-Value	Firth Penalized Logistic Regression aOR (95% CI)	*p*-Value
EGF treatment	39.43 (8.17–190.57)	<0.001	27.52 (7.86–146.85)	<0.001
Age (per 1-year increase)	0.94 (0.89–1.00)	0.040	0.94 (0.89–1.00)	0.039
HbA1c	1.00 (0.76–1.31)	0.991	1.00 (0.77–1.30)	0.986
Unsuccessful vs. successful revascularization	0.19 (0.05–0.78)	0.021	0.22 (0.05–0.80)	0.021
Partial vs. successful revascularization	0.48 (0.13–1.72)	0.255	0.50 (0.14–1.68)	0.259

Firth model statistics: Likelihood ratio test = 62.73, *p* < 0.001; *n* = 129. Abbreviations: aOR, adjusted odds ratio; CI, confidence interval; EGF, epidermal growth factor.

## Data Availability

The datasets generated and/or analyzed during the current study are available from the corresponding author upon reasonable request. The data are not publicly available due to privacy and ethical restrictions.

## Supplementary Materials

The following supporting information can be downloaded at: https://www.mdpi.com/article/10.3390/medicina62091800/s1. Figure S1: Gating strategy for flow cytometry.

## Abbreviations

The following abbreviations are used in this manuscript:

DF	diabetic foot
DFU	diabetic foot ulcer
iEGF	intralesional epidermal growth factor
EGF	epidermal growth factor
NPWT	negative pressure wound therapy
HBOT	hyperbaric oxygen therapy
PEDIS	Perfusion, Extent, Depth, Infection, and Sensation
LRINEC	Laboratory Risk Indicator for Necrotizing Fasciitis
WBC	white blood cell
CRP	C-reactive protein
HbA1c	glycated hemoglobin
ASA	American Society of Anesthesiologists
PBS	phosphate-buffered saline
M1	classically activated macrophage
M2	alternatively activated macrophage
ROS	reactive oxygen species
TNF-α	tumor necrosis factor-alpha
IL-1β	interleukin-1 beta
IL-6	interleukin-6
IL-10	interleukin-10
TGF-β	transforming growth factor-beta
aOR	adjusted odds ratio
OR	odds ratio
CI	confidence interval
VIF	variance inflation factor
IWGDF	International Working Group on the Diabetic Foot
IDSA	Infectious Diseases Society of America
FACS	fluorescence-activated cell sorting
