# Supplementary material for: Intralesional Epidermal Growth Factor and Its Association with Reamputation and Level-Up Amputation in Diabetic Foot Patients with Post-Amputation Flap Necrosis: Clinical Outcomes and Macrophage Dynamics
Source: Medicina (Kaunas). 2026 Sep 18;62(9):1800. doi: 10.3390/medicina62091800 (PMC13609649; doi:10.3390/medicina62091800)
Supplement: Supplementary file 1 [file medicina-62-01800-s001.zip › medicina-4547813-supplementary.pdf]

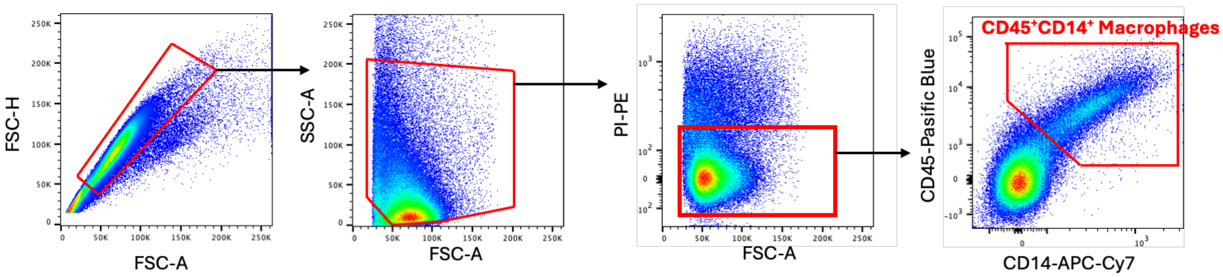

Figure S1. Gating strategy for flow cytometry. Doublets were excluded using FSC-H versus FSC-A. Healthy, non-apoptotic cells were then identified using SSC-A versus FSC-A. Propidium iodide (PI)-negative viable cells were subsequently selected for further analysis. Finally, all macrophage-associated marker analyses (CD38 and CD209 expression) were performed within the gated $CD45^{+}CD14^{+}$ macrophage population.
